# Supplementary material for: Initial whole-genome sequencing and analysis of the host genetic contribution to COVID-19 severity and susceptibility
Source: Cell Discov. 2020 Nov 10;6:83. doi: 10.1038/s41421-020-00231-4 (PMC7653987; doi:10.1038/s41421-020-00231-4)
Supplement: Supplementary file 1 — Supplementary materials [file 41421_2020_231_MOESM1_ESM.pdf]

Supplementary information

Initial Study of Human Genetic Contribution to COVID-19 Severity and Susceptibility

Fang Wang[1]*, Shujia Huang[2,3]*, Rongsui Gao[1]*, Yuwen Zhou[2,4]*, Changxiang Lai[1]*, Zhichao Li[2,4]*, Wenjie Xian[1], Xiaobo Qian[2,4], Zhiyu Li[1], Yushan Huang[2,4]， Qiyuan Tang[1], Panhong Liu[2,4], Ruikun Chen[1], Rong Liu[2], Xuan Li[1], Xin Tong[2], Xuan Zhou[1], Yong Bai[2], Gang Duan[1], Tao Zhang[2], Xun Xu[2,5], Jian Wang[2,6], Huanming Yang[2,6], Siyang Liu[2#], Qing He[1#], Xin Jin[2,3#], Lei Liu[1#]

1. The Third People's Hospital of Shenzhen, National Clinical Research Center for Infectious Disease, The Second Affiliated Hospital of Southern University of Science and Technology, Shenzhen 518112, Guangdong, China
2. BGI-Shenzhen, Shenzhen 518083, Guangdong, China
3. School of Medicine, South China University of Technology, Guangzhou 510006, Guangdong, China
4. BGI Education Center, University of Chinese Academy of Sciences, Shenzhen 518083, Guangdong, China
5. Guangdong Provincial Key Laboratory of Genome Read and Write，BGI-Shenzhen, Shenzhen, 518120，China
6. James D. Watson Institute of Genome Science, 310008 Hangzhou, China

*Those authors contribute equally

Corresponding to any of the followings:

Lei Liu liuleiszsdsrmyy@163.com

Xin Jin jinxin@genomics.cn

Qing He heqingjoe@163.com

Siyang Liu liusiyang@genomics.cn

Supplementary Figure

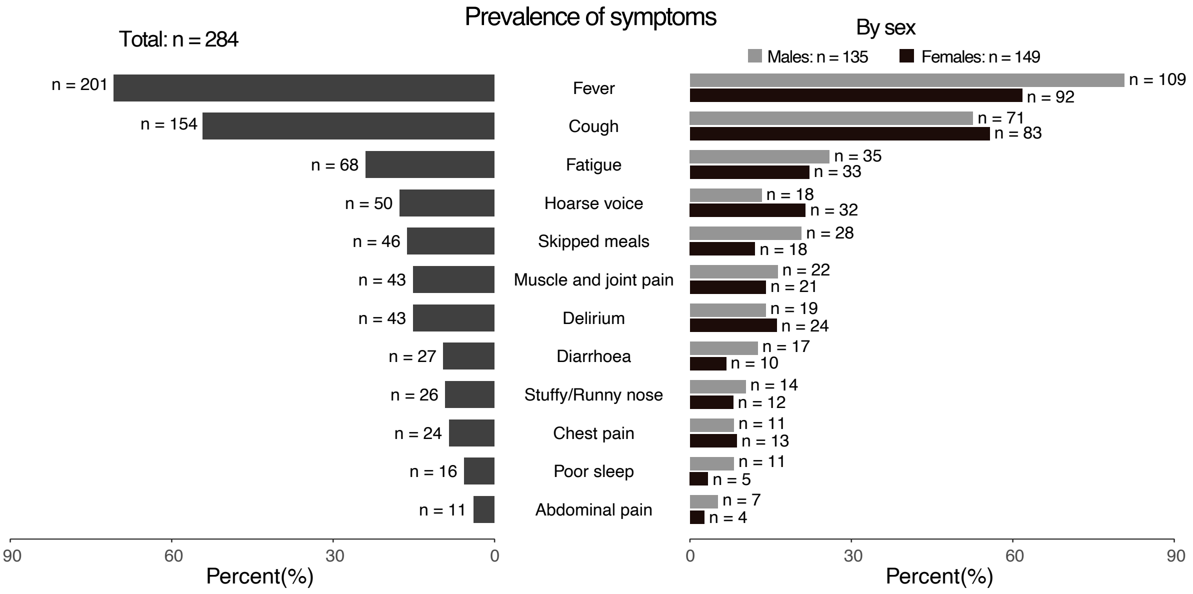

Supplementary Fig. S1. Clinical manifestation of the 284 unrelated individuals. Statistics were calculated from the patients' complaints in the electronic health records.

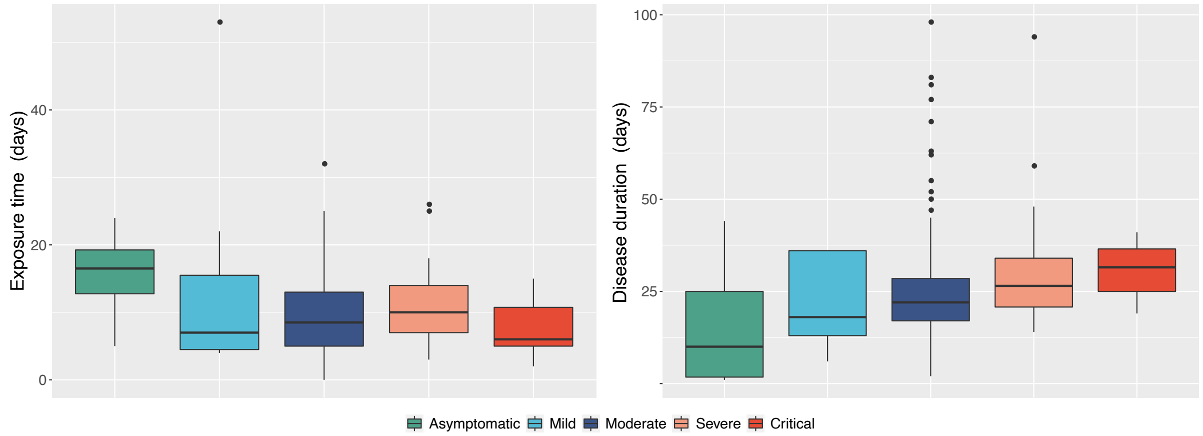

Supplementary Fig. S2. Exposure time and disease duration of the 332 patients by five categories.

A) Exposure time is defined as the time duration between the oral report of the first infected contact and the disease onset. B) Disease duration is defined as the time duration between disease onset and the first negative PCR-test.

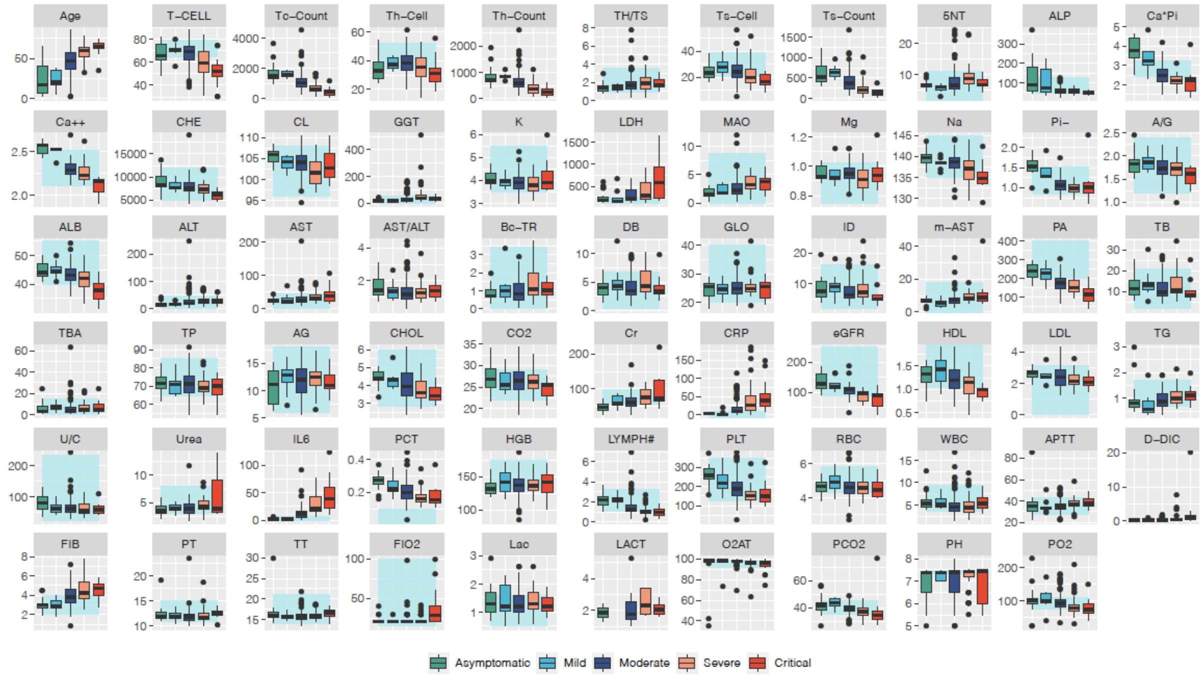

Supplementary Fig. S3. Distribution of age and sixty-four clinical laboratory assessments for the five groups of patients. Light blue boxes indicate the baseline of the healthy population in the electronic health records. The sixty-four laboratory belongs to the seven categories 1) blood count and blood chemical analysis (PLT, HGB, LYMPH#,RBC,WBC) 2) assessments of liver function (A/G, ALB, ALT, AST, AST/ALT, Bc-TR, DB, GLO, ID, m-AST, PA, TB, TBA, T), 3) assessments of renal function (AG, CHOL, CO2, Cr, CRP, eGFR, HDL, LDL, TG, U/C, Urea), 4) tests of humoral immunity (PCT, IL6), 5) tests of coagulation (APTT, D-DIC, FIB, PT, TT) and 6) measures of electrolyte (5NT, ALP, Ca*Pi, Ca++, CHE, CL, GGT, K, LDH, MAO, Mg, Na, Pi-) and 7) blood gas electrolyte (FIO2, Lac, O2AT, PCO2, PH, PO2, LACT).

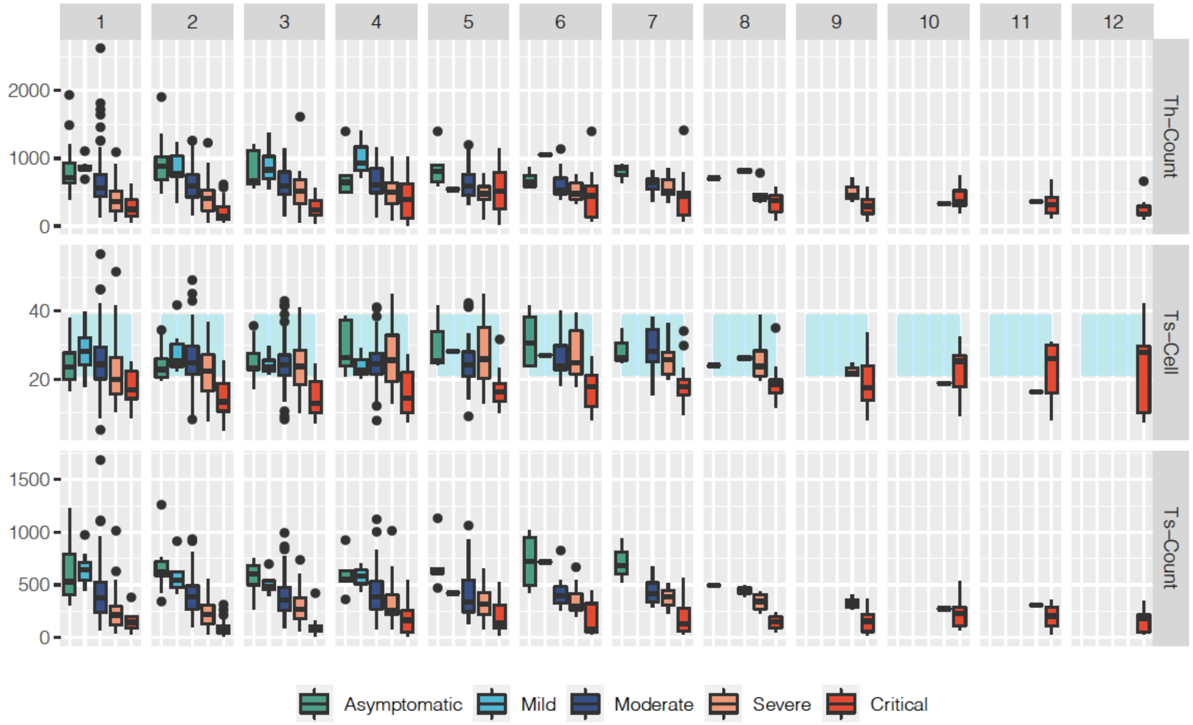

Supplementary Fig. S4. Distribution of the T lymphocyte subgroups for the five disease categories as a function of time ranging from the first to the twelfth assessment.

284 unrelated patients

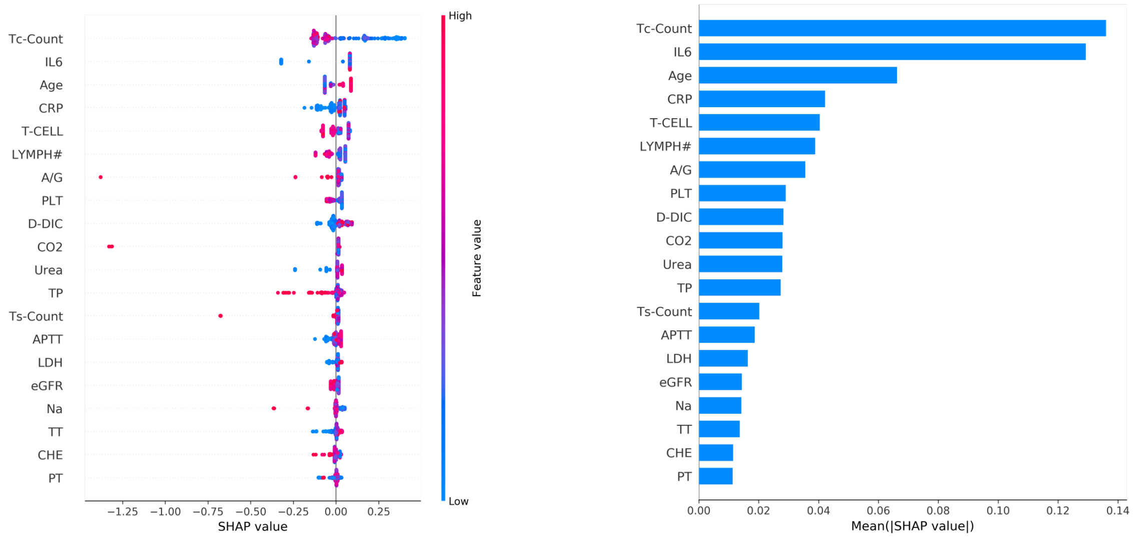

332 patients

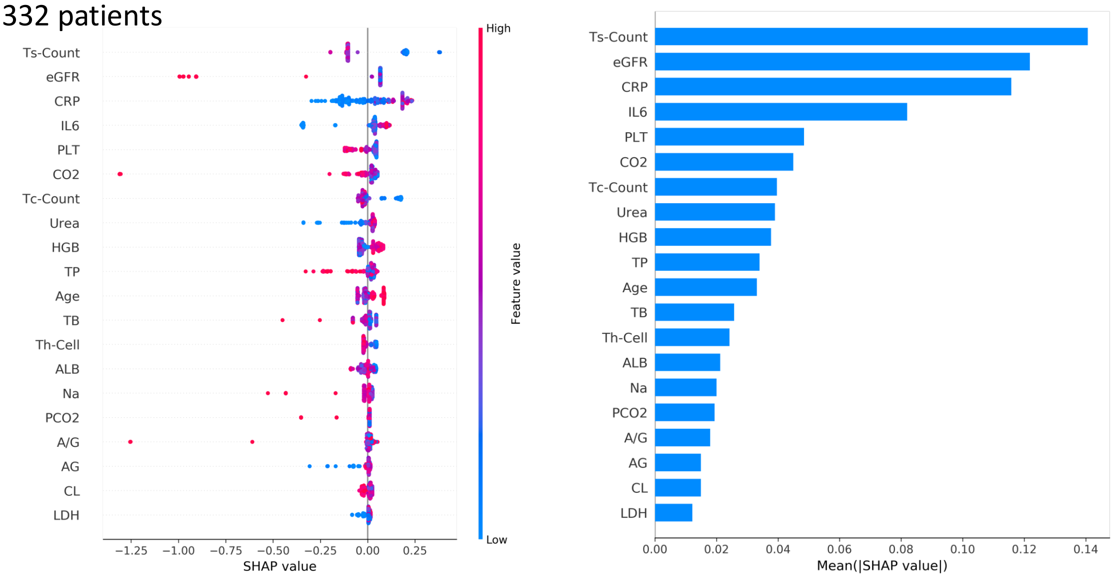

Supplementary Fig. S5. Importance of the sixty four laboratory assessment features to classify the five groups of patients. Shown are the top twenty features of the greatest importance. Shown are the distribution of the SHAP prediction value (which indicates the effect of each feature on the classification of the patient severity) for each laboratory assessment feature (y-axis) for each patient (each dot). Minus and positive values indicate negative and positive effects. Top: distribution for the 284 unrelated patients. Bottom: distribution for all the 332 patients.

284 unrelated patients

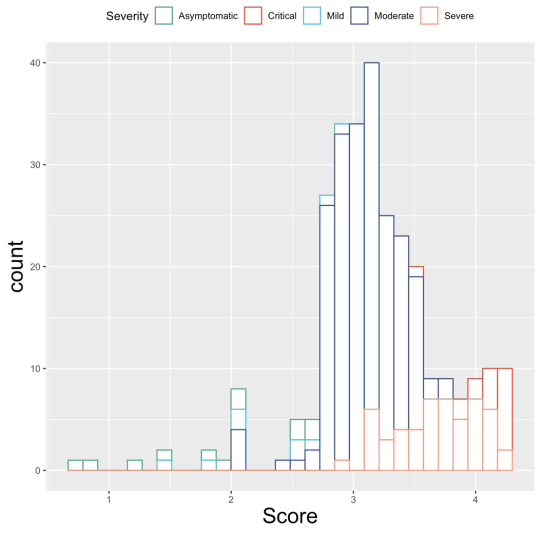

332 patients

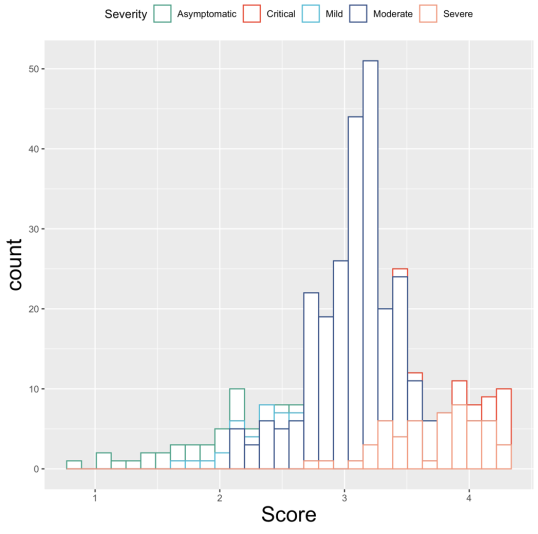

Supplementary Fig. S6. Distribution of the severity score according to the five severity categories

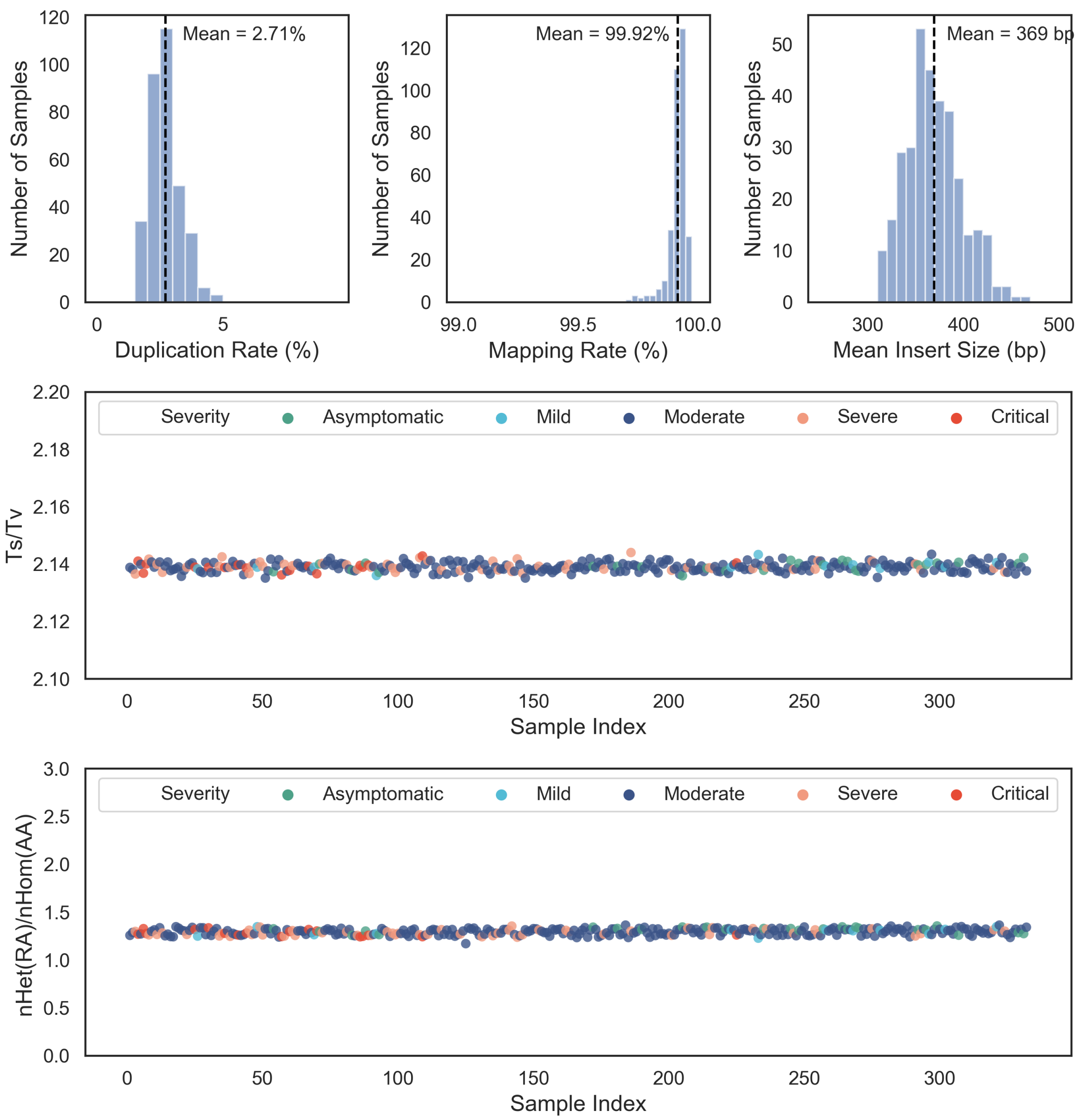

Supplementary Fig. S7. Quality control of individual sequenced genome

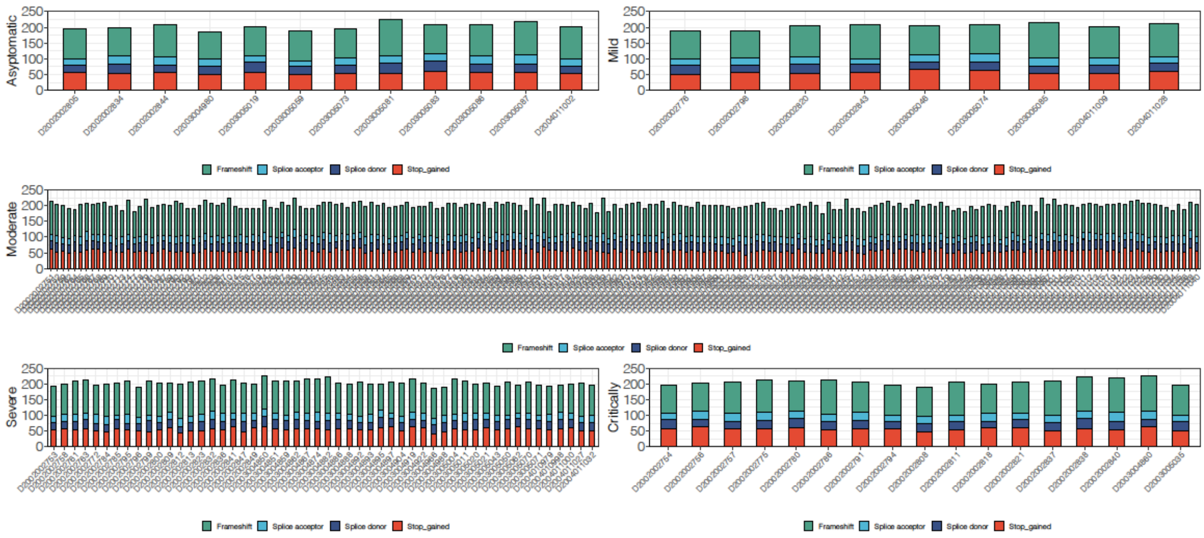

Supplementary Fig. S8. Estimated number of loss of function variants for each patient. The label of the y-axis indicates the asymptomatic, mild, moderate, severe and critical group, respectively. The label of the x-axis indicate sample ID of the patient. Frameshift, splice acceptor, splice donor and stop gain variants are shown in green, skype blue, dark blue and red, respectively.

Tissue	Samples	NES	p-value	m-value
Brain - Spinal cord (cervical c-1)	126	0.0463	0.9	0.834
Stomach	324	0.0153	0.9	0.434
Cells - EBV-transformed lymphocytes	147	-0.0794	0.8	0.947
Small Intestine - Terminal Ileum	174	-0.0941	0.6	0.619
Ovary	167	-0.113	0.7	0.938
Prostate	221	-0.184	0.3	0.878
Brain - Nucleus accumbens (basal ganglia)	202	-0.190	0.3	0.801
Minor Salivary Gland	144	-0.199	0.3	0.882
Brain - Caudate (basal ganglia)	194	-0.210	0.3	0.938
Brain - Cerebellum	209	-0.225	0.3	0.906
Colon - Transverse	368	-0.295	0.006	0.992
Brain - Cortex	205	-0.305	0.1	0.972
Brain - Hippocampus	165	-0.307	0.2	0.967
Skin - Not Sun Exposed (Suprapubic)	517	-0.329	0.02	0.993
Brain - Cerebellar Hemisphere	175	-0.341	0.08	0.972
Spleen	227	-0.341	0.05	0.958
Artery - Tibial	584	-0.351	0.007	1.00
Brain - Anterior cingulate cortex (BA24)	147	-0.377	0.3	0.945
Whole Blood	670	-0.400	1.8e-10	1.00
Adipose - Visceral (Omentum)	469	-0.418	4.4e-3	1.00
Esophagus - Mucosa	497	-0.451	2.2e-3	1.00
Heart - Left Ventricle	386	-0.477	6.3e-4	1.00
Brain - Putamen (basal ganglia)	170	-0.478	0.03	0.985
Brain - Frontal Cortex (BA9)	175	-0.491	0.03	0.978
Colon - Sigmoid	318	-0.500	0.006	0.997
Uterus	129	-0.505	0.2	1.00
Pancreas	305	-0.520	3.3e-3	1.00
Skin - Sun Exposed (Lower leg)	605	-0.525	1.8e-5	1.00
Esophagus - Gastroesophageal Junction	330	-0.527	0.01	1.00
Breast - Mammary Tissue	396	-0.528	3.0e-3	1.00
Esophagus - Muscularis	465	-0.540	1.5e-4	1.00
Vagina	141	-0.542	0.1	1.00
Nerve - Tibial	532	-0.551	3.4e-5	1.00
Pituitary	237	-0.573	0.2	0.931
Muscle - Skeletal	706	-0.651	2.5e-8	1.00
Lung	515	-0.669	7.6e-8	1.00
Adipose - Subcutaneous	581	-0.678	9.1e-8	1.00
Heart - Atrial Appendage	372	-0.679	1.8e-5	1.00
Testis	322	-0.692	1.6e-3	1.00
Adrenal Gland	233	-0.704	4.2e-4	1.00
Thyroid	574	-0.762	2.8e-8	1.00
Artery - Coronary	213	-0.762	0.01	0.991
Cells - Cultured fibroblasts	483	-0.769	2.1e-7	1.00
Brain - Substantia nigra	114	-0.792	0.01	1.00
Brain - Hypothalamus	170	-0.954	3.5e-4	1.00
Liver	208	-1.13	3.0e-3	0.999
Artery - Aorta	387	-1.21	7.8e-10	1.00
Kidney - Cortex	73	-1.36	0.04	0.976
Brain - Amygdala	129	-1.62	2.5e-4	1.00

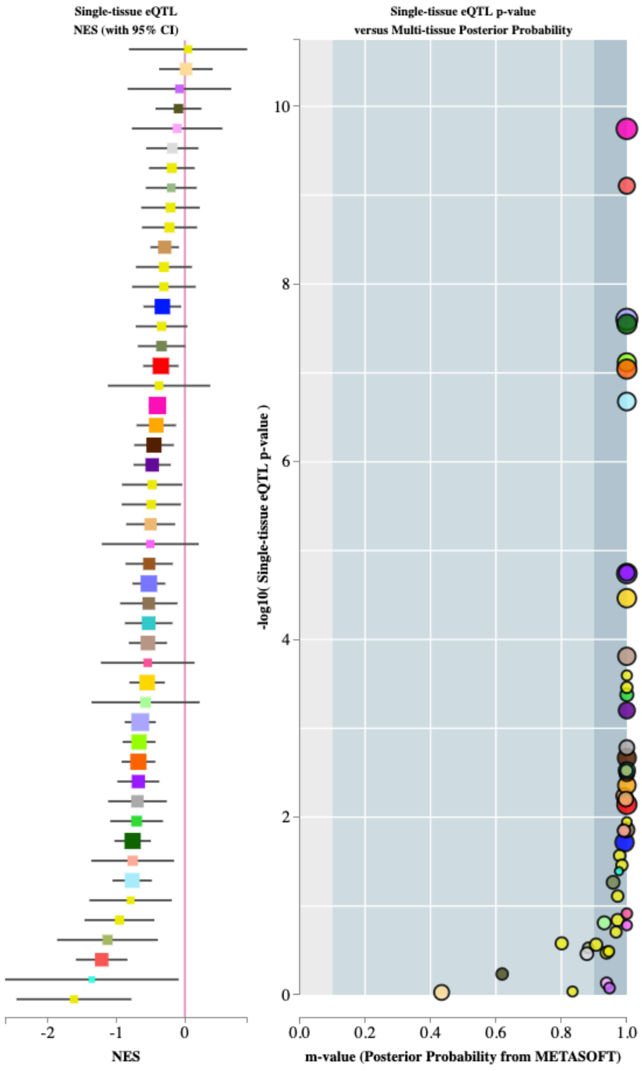

Supplementary Fig. S9. Decreased expression of DPP7 in multiple tissues given the loss of function insertion (rs11391519) present in two asymptomatic patients.

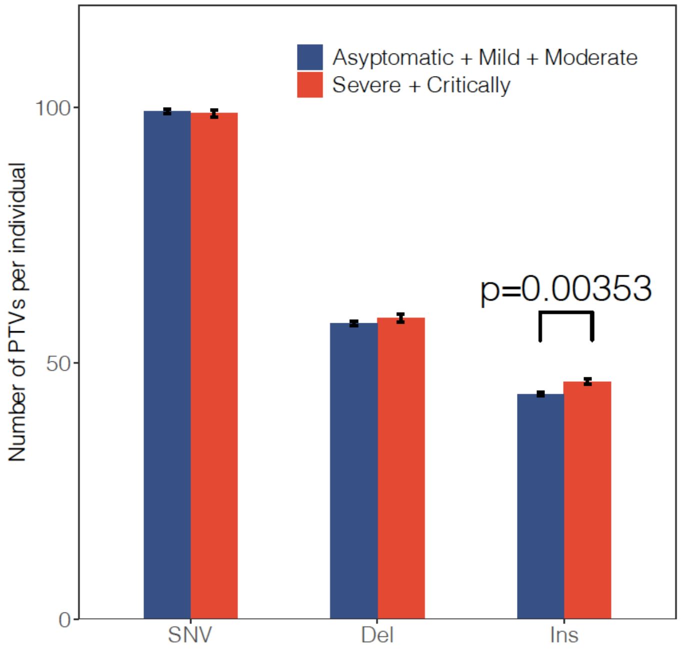

Supplementary Fig. S10. Comparison of loss of function variation burden for SNP, small insertions and deletions between the severe and the non-severe patients.

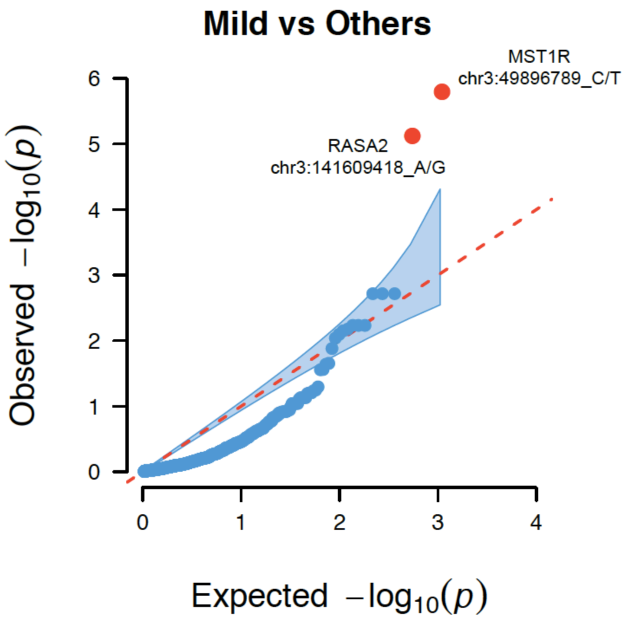

Supplementary Fig. S11. Loss of function variants between the asymptomatic and mild groups of patients versus the rest of the patients.

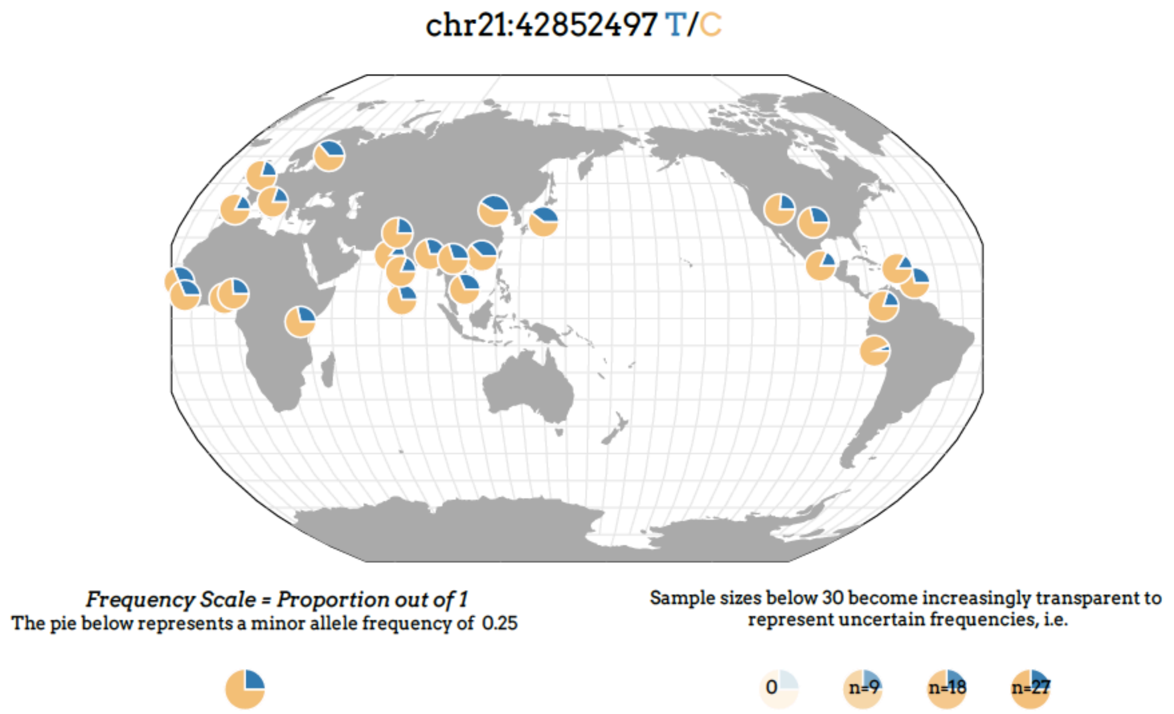

Supplementary Fig. S12. Allele frequency of the p.Val197Met variant in TMPRSS2 among the 1000 genomes populations. The allele frequency of the reference and alternative allele is visualized by the geography of genetic variants browser developed by the university of Chicago. p.Val197Met is located at the 42852497 position in chr21 with rsID rs12329760.

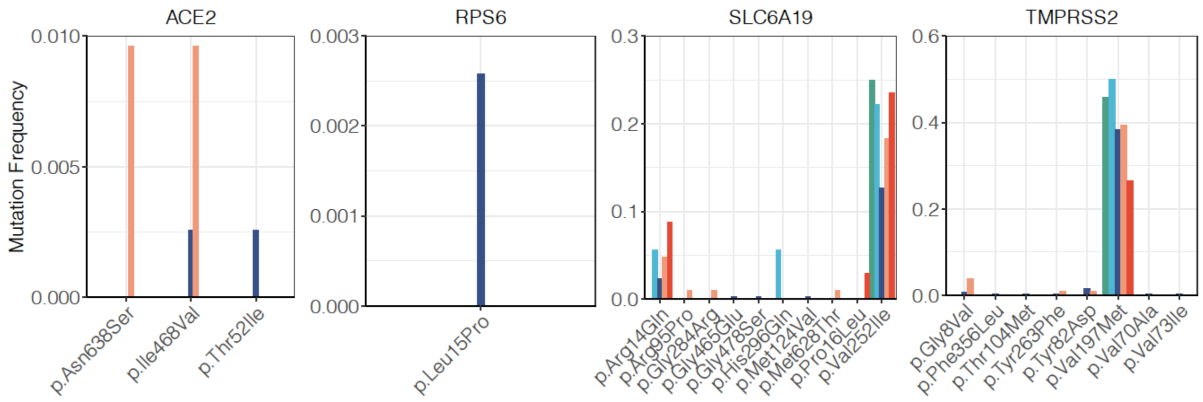

Supplementary Fig. S13. Allele frequency distribution for the functional variants present in nine genes related to the host-pathogen interaction by Sharma et al., 2020 BioRxiv among the five patient severity groups. Genes *ADAM17*, *HNRNPA1*, *SUMO1*, *NACA* and *BTF3* doesn't contain any missense and loss of function variants among the patients. Shown are the allele frequency of the missense variants found in *ACE2*, *PRS6*, *SLC6A19* and *TMPRSS2* among the five patient group. No loss of function variants was present in those genes.

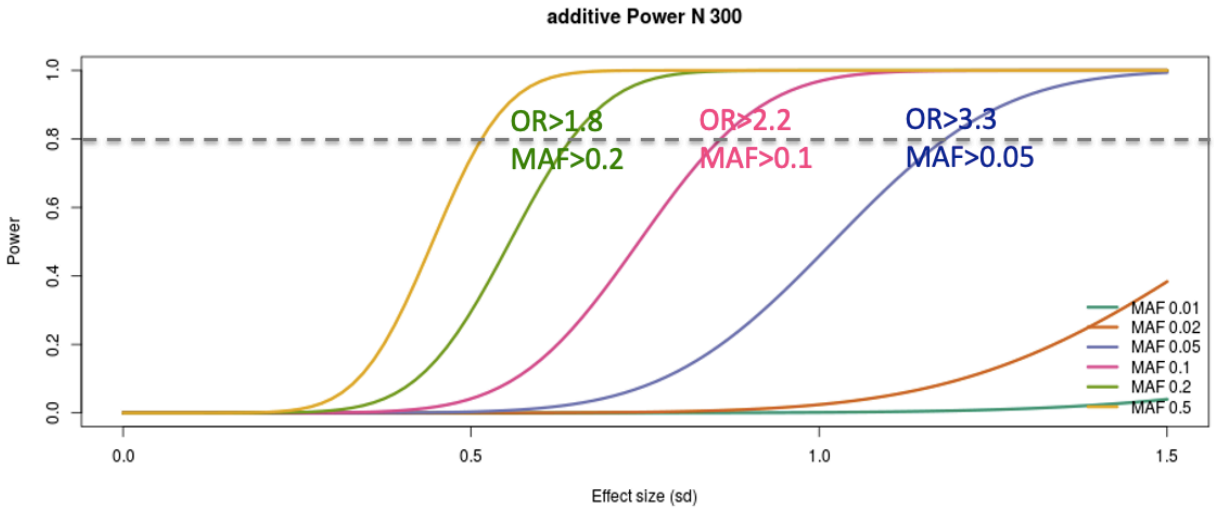

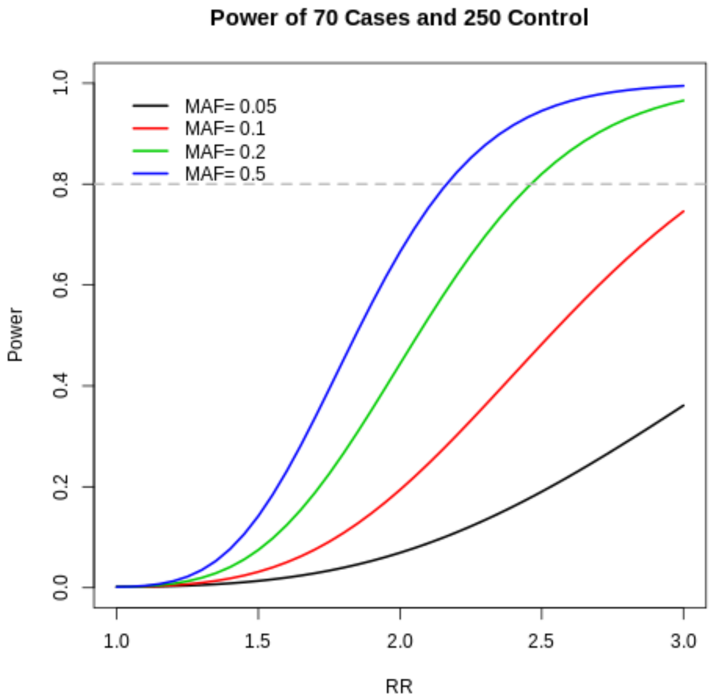

Supplementary Fig. S14. Power for single variant association test. Top: linear regression Bottom: Logistic regression. Significant threshold=1e-7

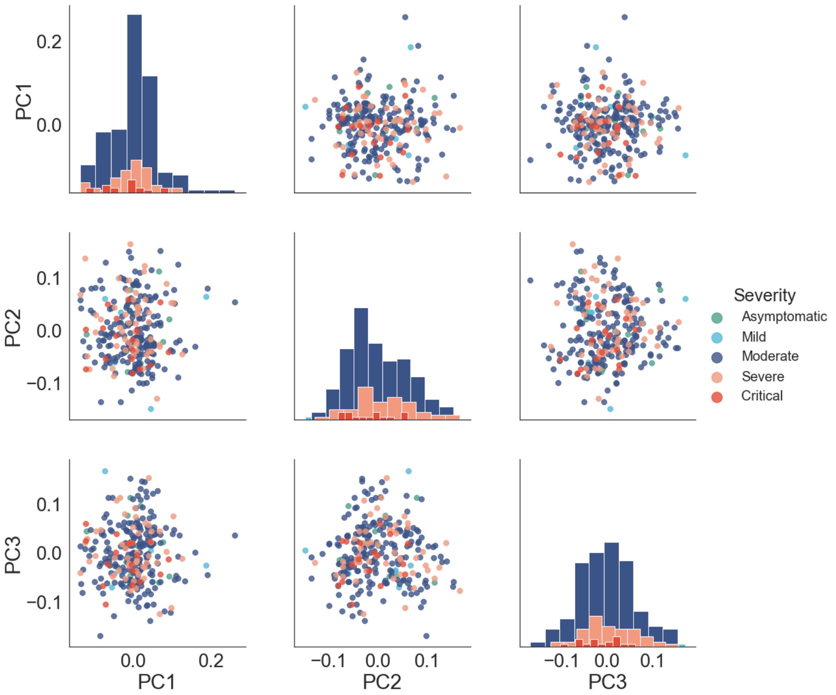

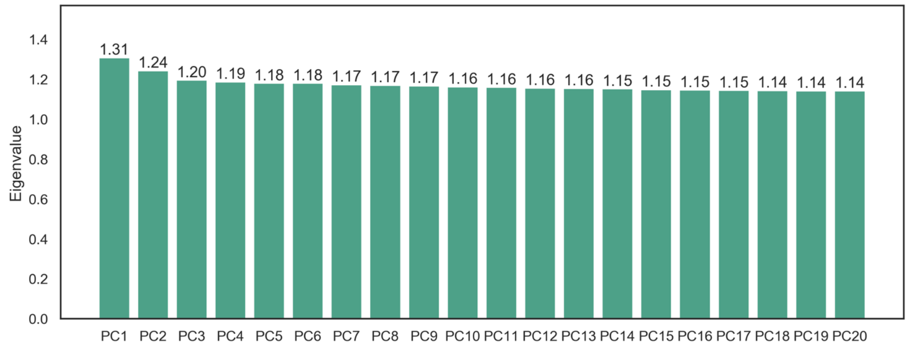

Supplementary Fig. S15. Principle component analysis of the unrelated COVID-19 patients (N=284) recruited in the study. Shown are the top 3 eigenvectors and the top 20 eigenvalues for the principle component analysis using plink.

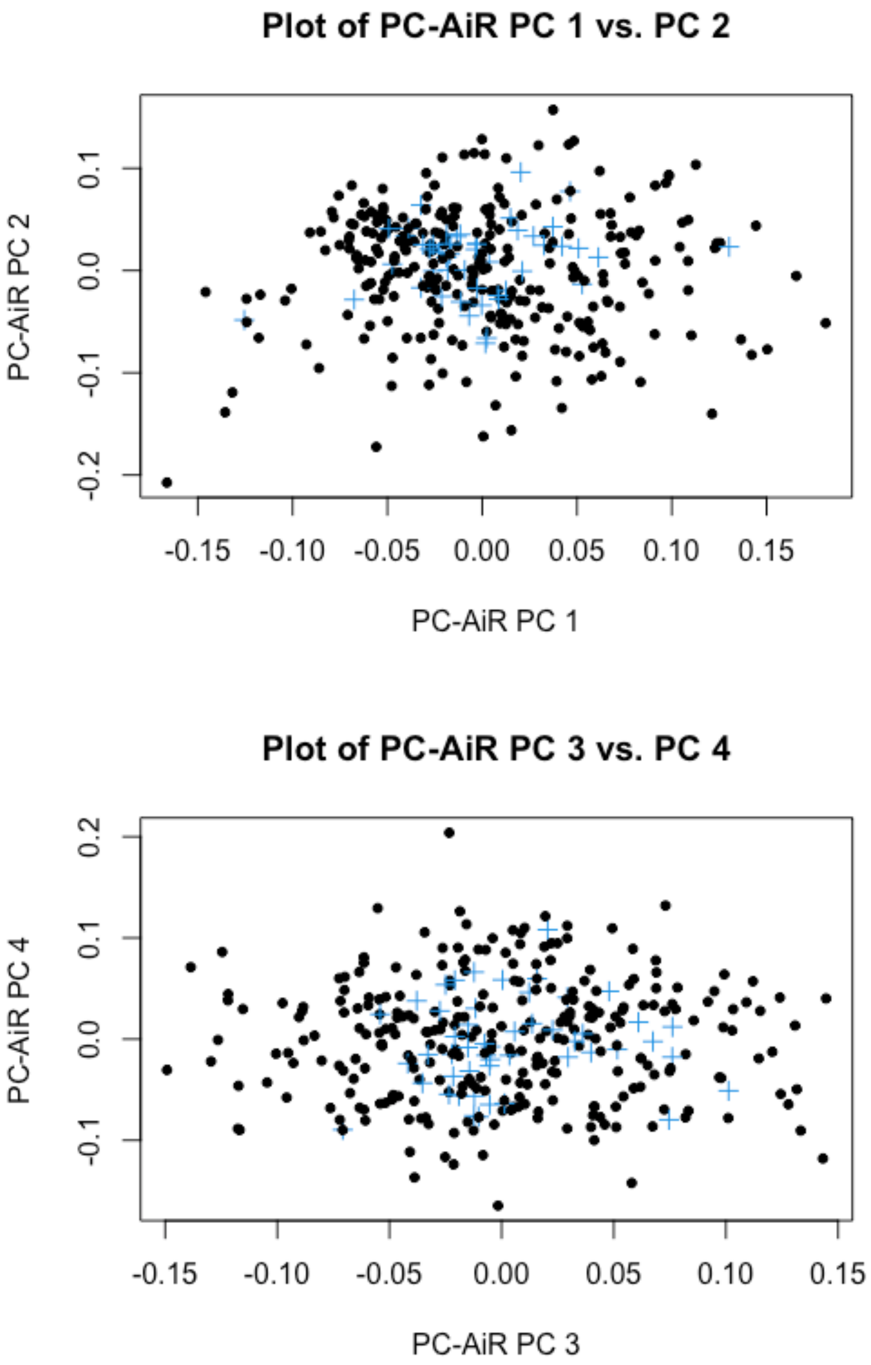

Supplementary Fig. S16. Principle component analysis of the all the COVID-19 patients (N=332) recruited in the study. Shown are the top 4 eigenvectors and the top 20 eigenvalues in the principle component analysis using PC-AiR in Genesis R package. Black dot indicates unrelated individual. Blue cross indicates related individuals.

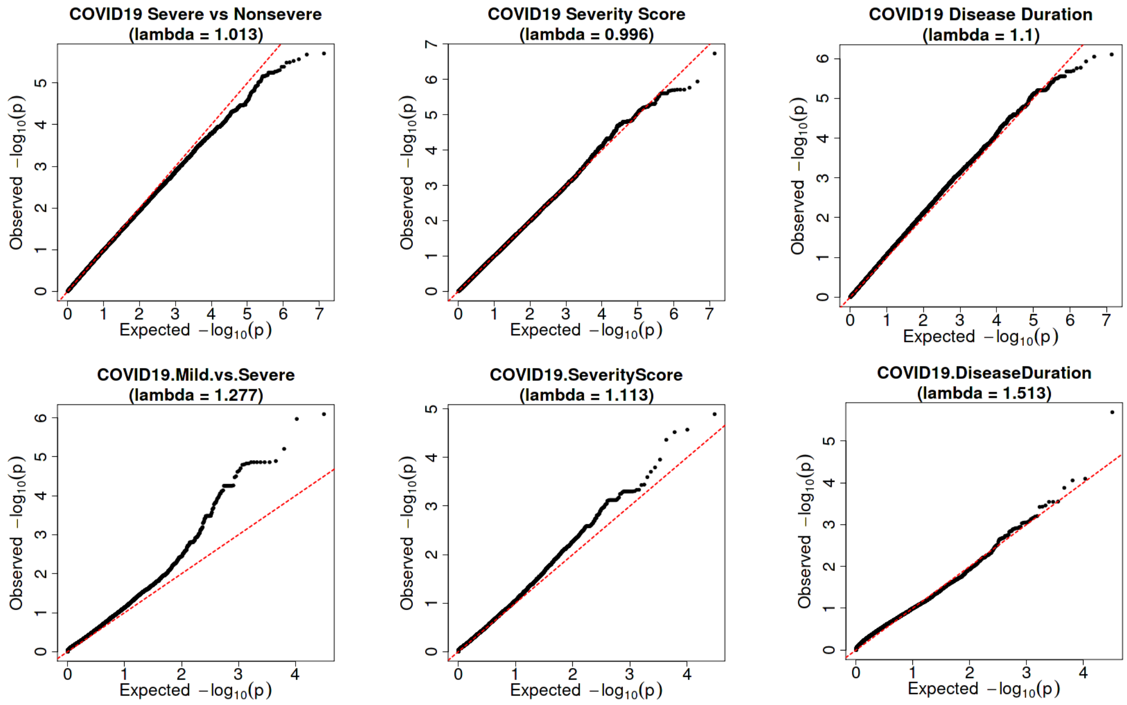

Supplementary Fig. S17. Quantile-quantile plots for the three traits, linked to Fig. 3.

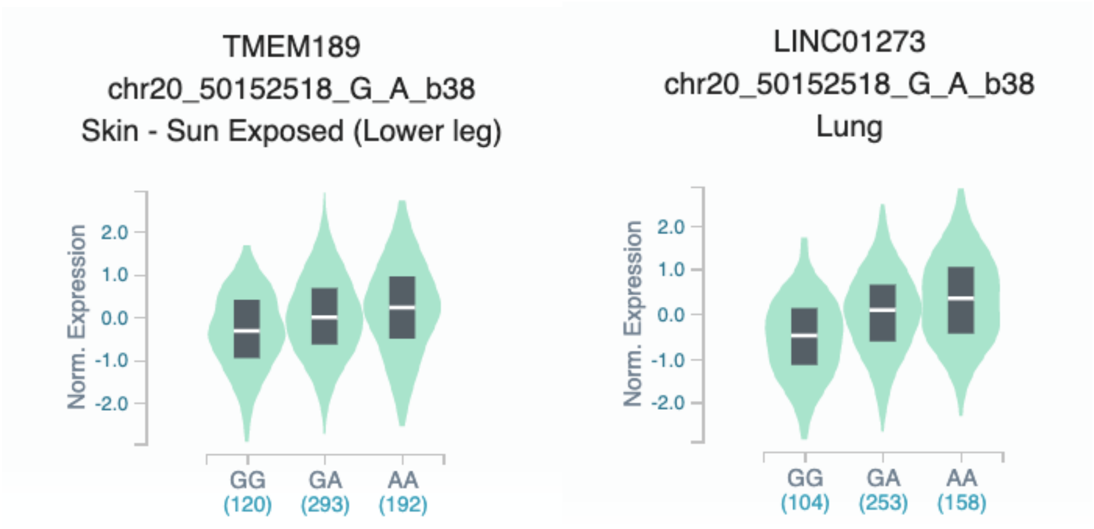

Supplementary Fig. S18. Altered gene expression given the three genotypes at the lead SNP rs6020298. Figure from Gtex portal using the GTEx Analysis Release V8. The A allele increases *LINC01273*, *TMEM189* expression over almost all the tissues. Only two representative plots were shown here.

COVID19_HGI_ANA_A2_V2_20200701.txt

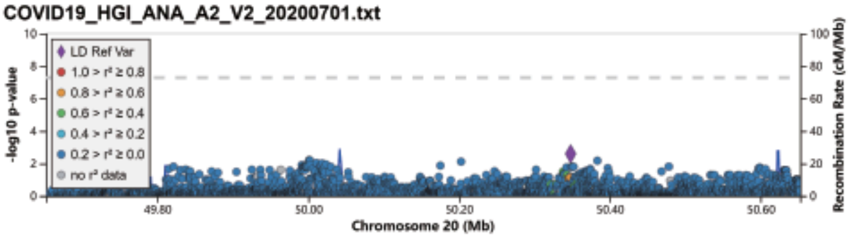

COVID19_HGI_ANA_B1_V2_20200701.txt

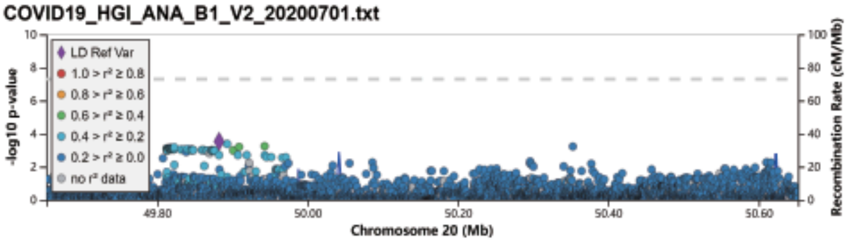

COVID19_HGI_ANA_B2_V2_20200701.txt

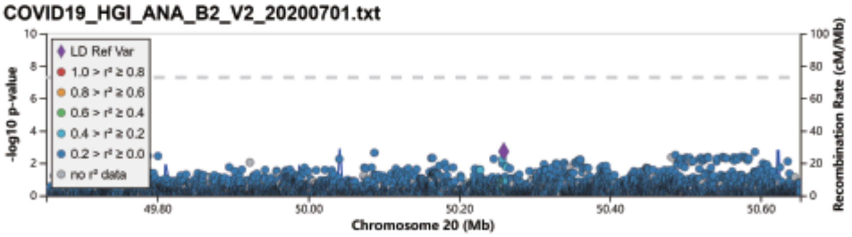

COVID19_HGI_ANA_C1_V2_20200701.txt

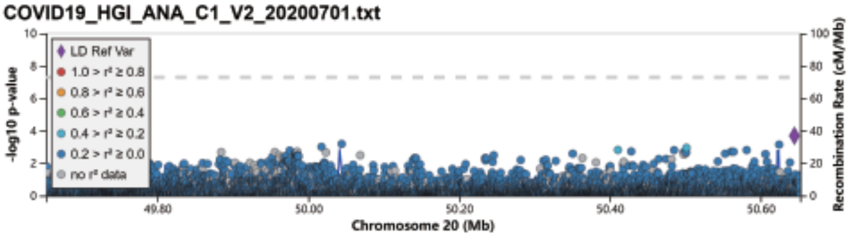

COVID19_HGI_ANA_C2_V2_20200701.txt

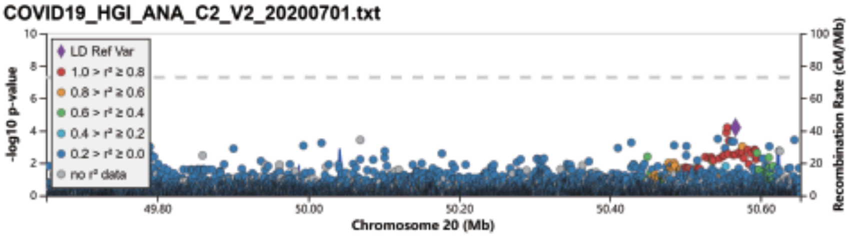

COVID19_HGI_ANA_D1_V2_20200701.txt

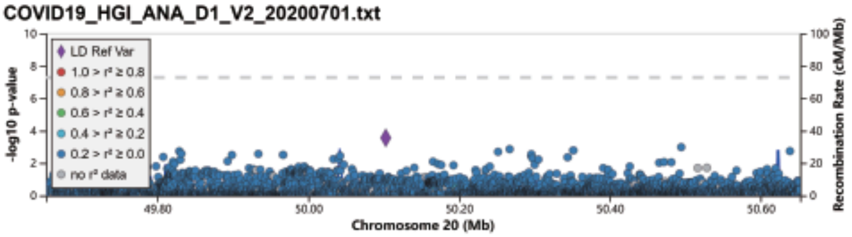

Supplementary Fig. S19. Locuszoom shows the p-value of lead SNP rs6020298 identified in our study using the host genetic initiative summary data (https://www.covid19hg.org/results/).

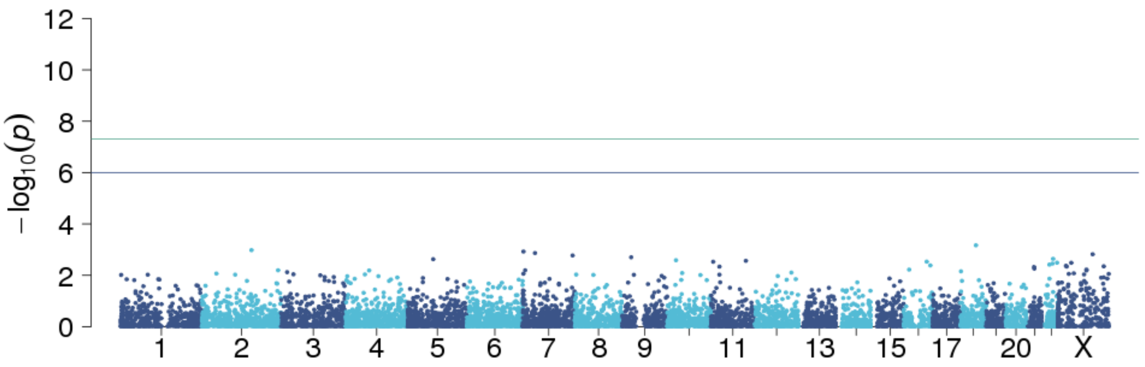

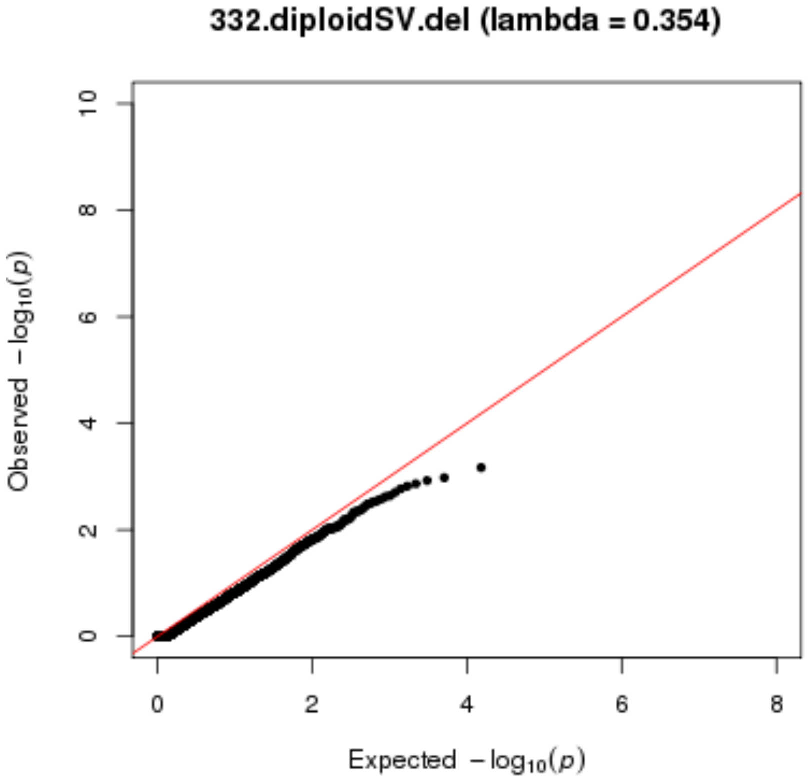

Supplementary Fig. S20. Genome-wide association tests for 81,193 copy number variations identified from the 332 individuals. Top: manhattan plot .Bottom: quantile-quantile plot

CXCR6

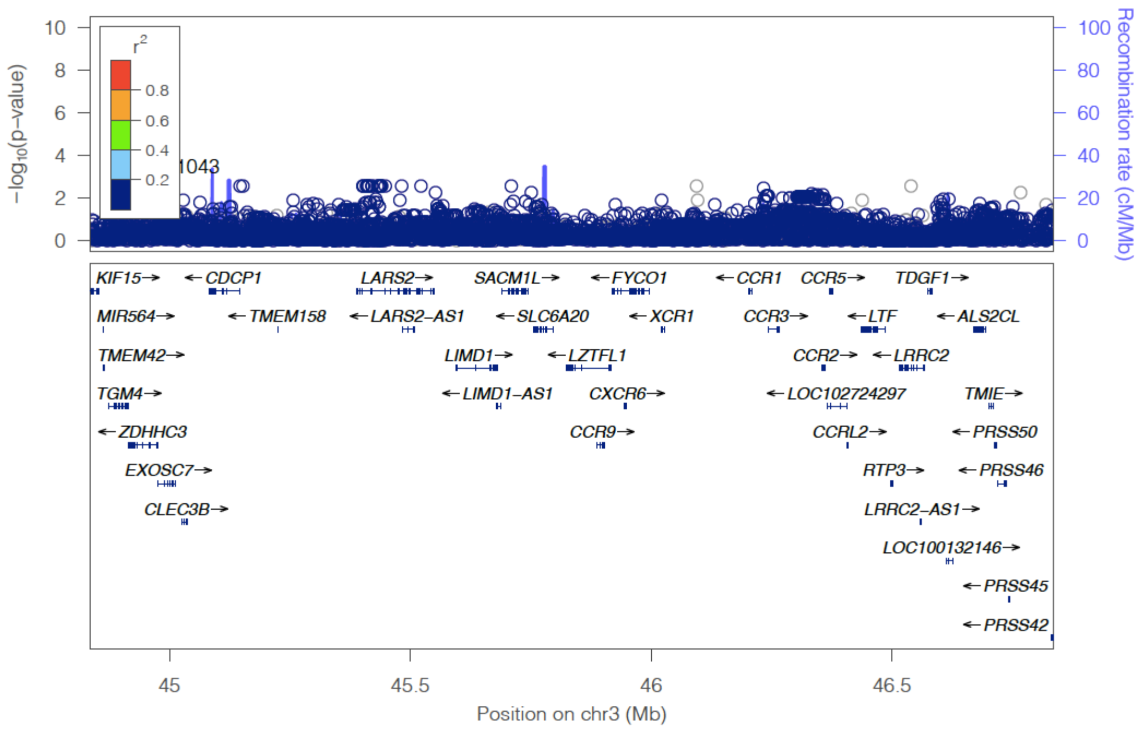

10
8
6
4
2
0
−log10(p−value)
r2
0.8
0.6
0.4
0.2
1043
100
80
60
40
20
0
Recombination rate (cM/Mb)
KIF15→
←CDCP1
LARS2→
SACM1L→
←FYCO1
←CCR1
CCR5→
TDGF1→
MIR564→
←TMEM158
←LARS2−AS1
←SLC6A20
←XCR1
CCR3→
←LTF
←ALS2CL
TMEM42→
LIMD1→
←LZTFL1
CCR2→
←LRRC2
TGM4→
←LIMD1−AS1
CXCR6→
←LOC102724297
TMIE→
←ZDHHC3
CCR9→
CCRL2→
←PRSS50
EXOSC7→
RTP3→
←PRSS46
CLEC3B→
LRRC2−AS1→
LOC100132146→
←PRSS45
←PRSS42
45
45.5
46
46.5
Position on chr3 (Mb)

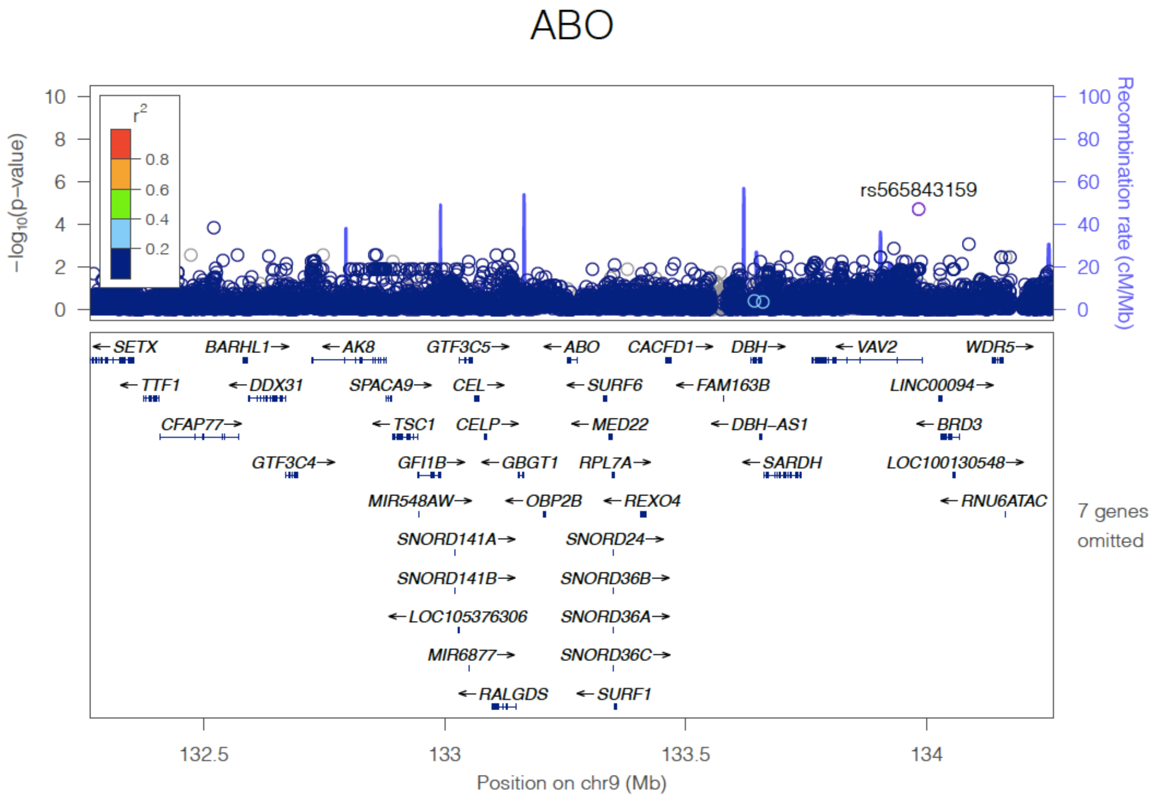

Supplementary Fig. S21. Locuszoom shows the p-value of the genome-wide association test between severe and non-severe patients for the two loci reported in “Genome wide association study of severe COVID-19 with respiratory failure”.

Top: lead snp rs11385942 on 3p21.31, coordinates on hg38 chr3: 45834967- 45834967.

Bottom: lead snp 133263862 on 9q34.2, coordinates on hg38 chr9: 133263862-133263862

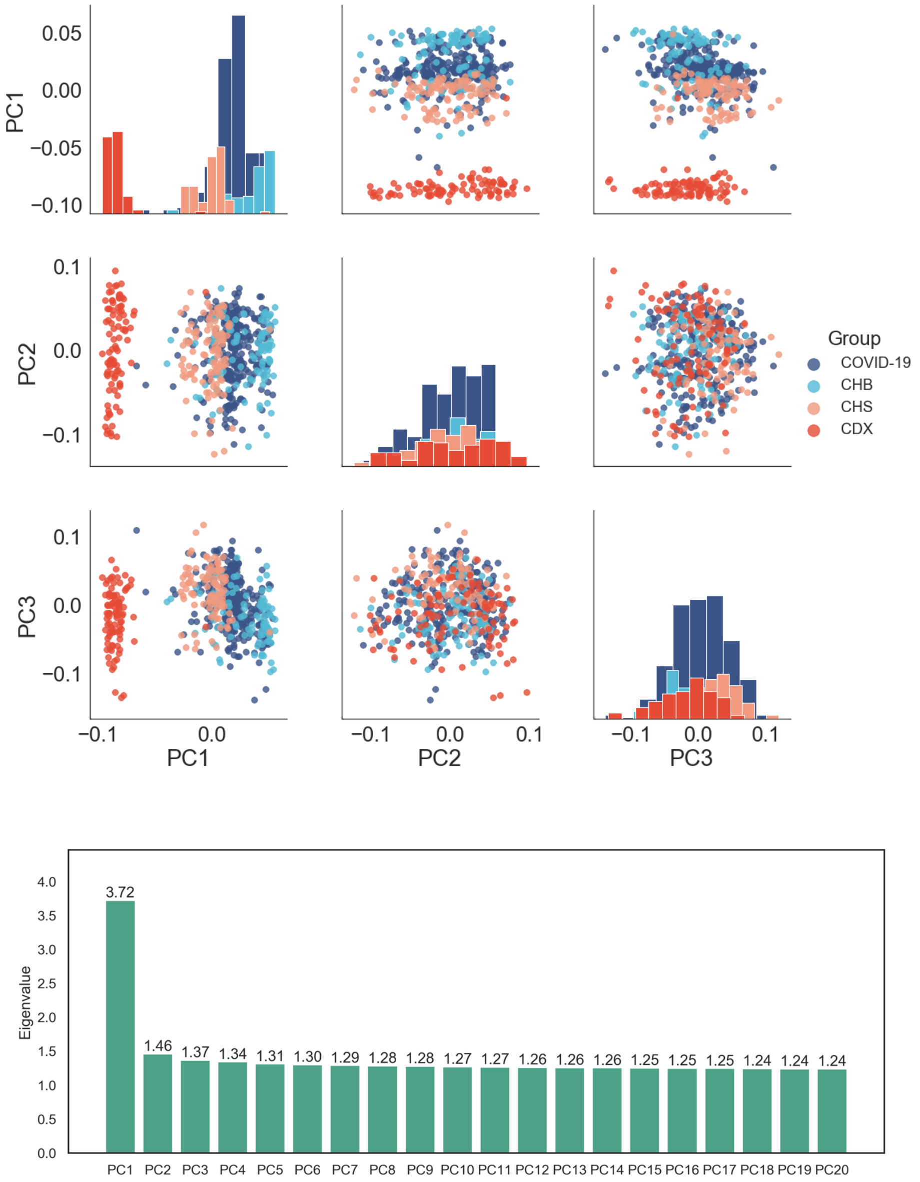

Supplementary Fig. S22. Principle component analysis for the unrelated patients (N=284) and the 1KGP Chinese population (N=301). CHB: Chinese from Beijing, CHS: Chinese from the South, CDX: Chinese Dai in Xishuangbanna, China.

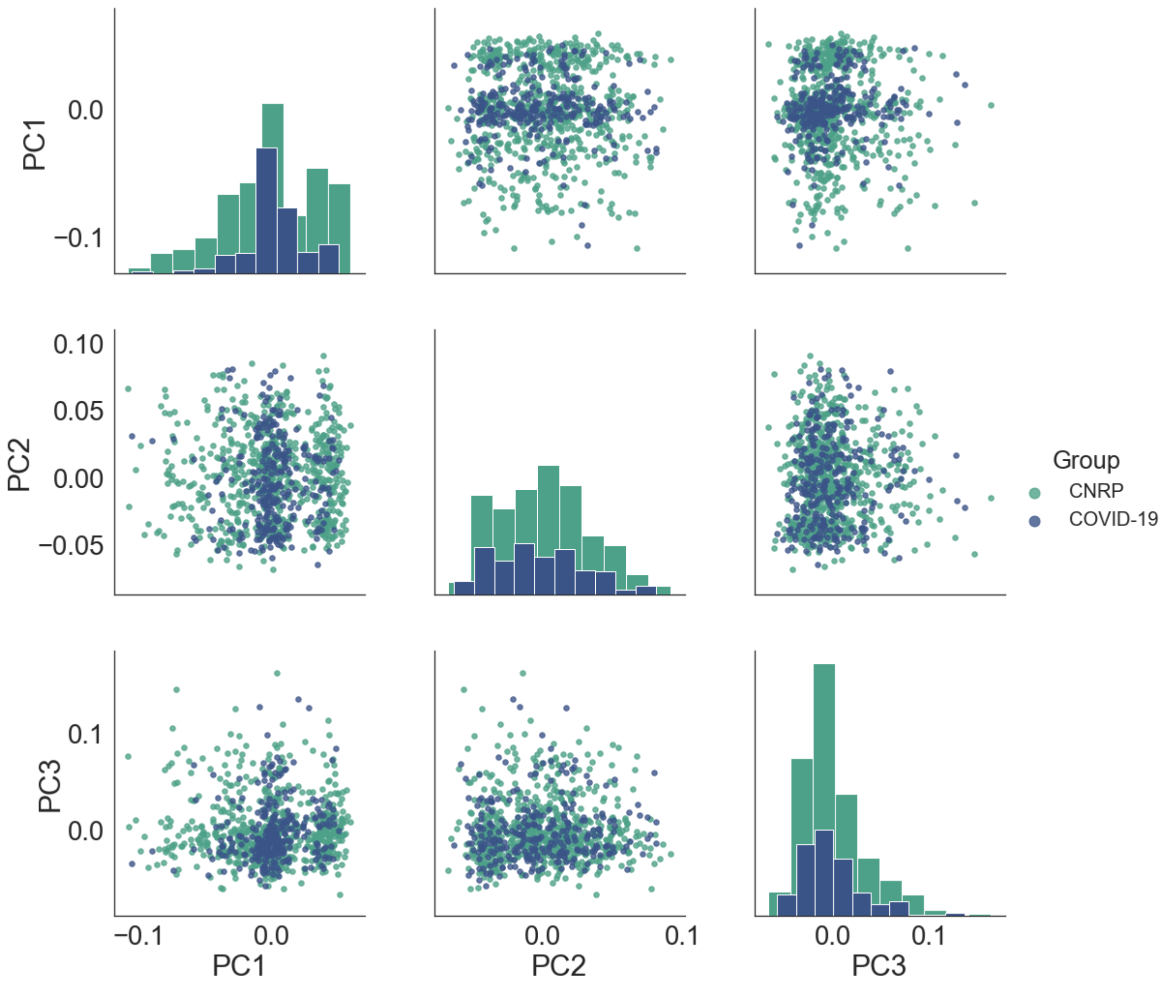

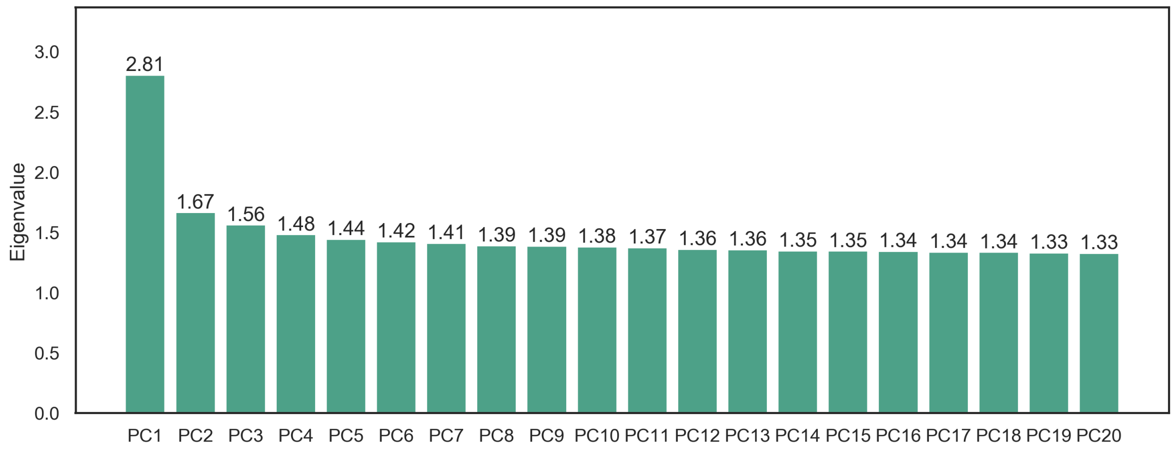

Supplementary Fig. S23. Principle component analysis for the 284 unrelated patients and the 665 control individuals in the Chinese Reference Panel program.

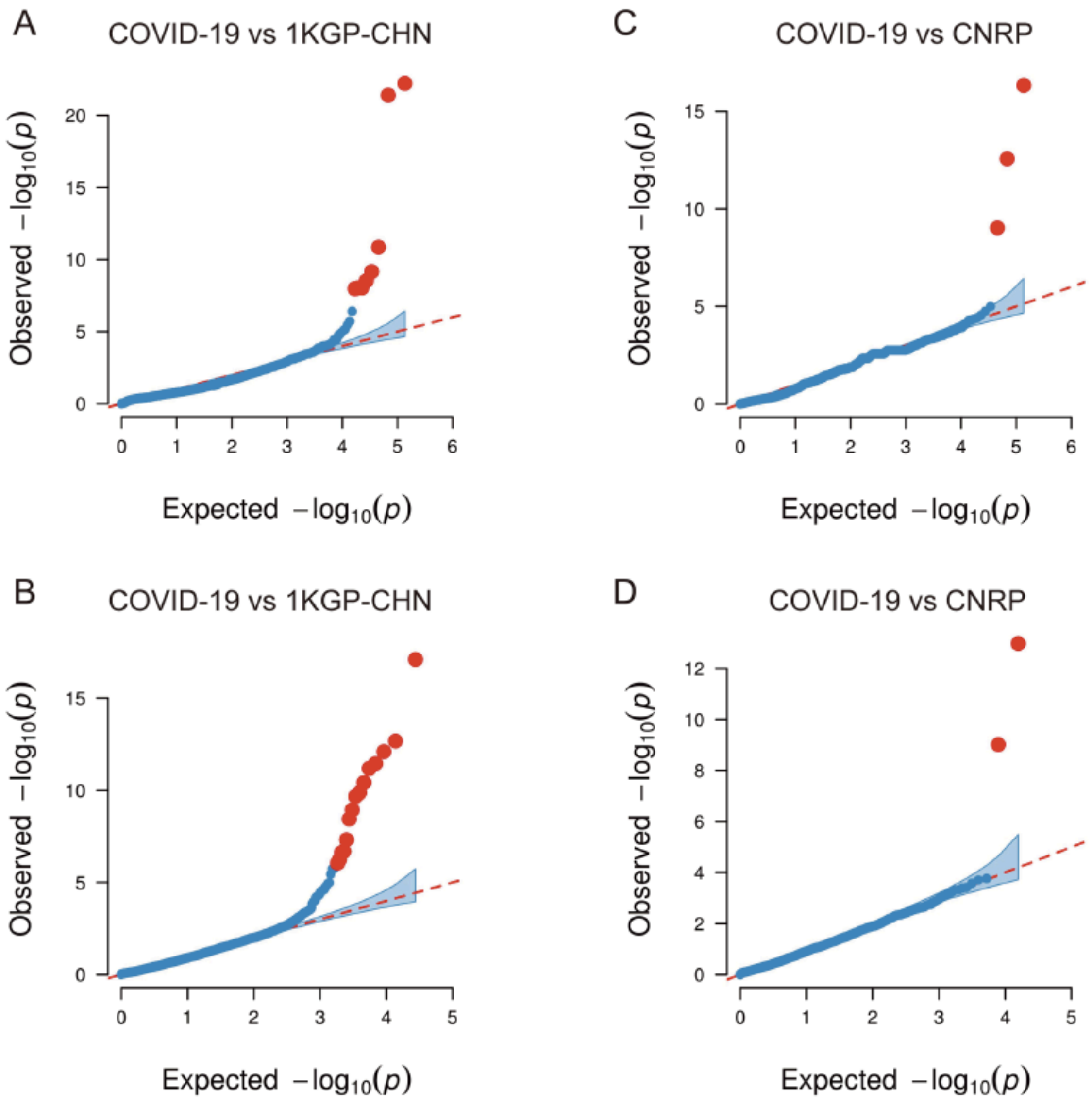

Supplementary Fig. S24. Quantile-quantile plot for single variant and gene-based association test between COVID-19 patients and the general populations. (A) single variant association test and (B) gene-based association test between the unrelated COVID-19 patients (N=284) and the 1KGP Chinese population (N=301) (C) single variant association test and (D) gene-based association test between the unrelated COVID-19 patients (N=284) and the CNRP Chinese population (N=271). Only variants with moderate or high impacts by variant effect predictor were shown in (A) and (C).
